# 适用于法庭科学毒物分析的干血斑检验体系的建立——以5种常见药(毒)物为例

**DOI:** 10.3724/SP.J.1123.2023.07035

**Published:** 2024-03-08

**Authors:** Dongbin SU, Linpei DONG, Yunfeng ZHANG, Peng ZHAO, Kaikai LI

**Affiliations:** 1.中国人民公安大学侦查学院, 北京 100038; 1. College of Investigation, People’s Public Security University of China, Beijing 100038, China; 2.公安部鉴定中心, 北京 100038; 2. Institute of Forensic Science, Ministry of Public Security, Beijing 100038, China

**Keywords:** 超高效液相色谱-串联质谱, 毒物分析, 干血斑, 储存条件, 稳定性, ultra-high performance liquid chromatography-tandem mass spectrometry (UHPLC-MS/MS), toxicant analysis, dried blood spots (DBS), storage conditions, stability

## Abstract

干血斑技术能够方便地对血液样品中的违禁药物进行快速分析,在酒后驾驶检查、滥用药物检测、兴奋剂检测等毒物分析场景具有显著优势。然而在我国法庭科学毒物分析领域,因缺少标准化检验体系,其稳定性和可靠性未得到深入研究论证,限制了其在司法实践中的运用。本研究以甲基苯丙胺、利多卡因、氯胺酮、芬太尼和地西泮为典型药(毒)物,使用整个干血斑进行分析,建立了适用于法庭科学领域毒物分析的超高效液相色谱-串联质谱分析方法,形成了以干血斑样品制作、前处理、分析、储存和效用性评价为主要内容的检验体系,并为干血斑中其他药(毒)物的分析方法开发提供参考。结果表明,干血斑中利多卡因和芬太尼在0.5~100 ng/mL内线性关系良好,甲基苯丙胺、氯胺酮、地西泮在2~100 ng/mL内线性关系良好,方法检出限为0.2~0.5 ng/mL。干血斑中5种目标物可以在60天内保持稳定,目标物测定含量与理论值的偏差在15%以内。干血斑中5种目标物的测量结果与全血一致,没有显著的系统误差和比例误差,芬太尼、地西泮、氯胺酮、利多卡因和甲基苯丙胺的测量浓度的相对偏差分别为4.44%、3.50%、7.66%、5.10%和5.25%。干血斑样品前处理方法简单,样品用量小,能够实现血液样品保存的轻量化和规范化且与全血样品具有高度定量一致性,可为公安实践工作中分析、保存血液检材提供新方案。

## Abstract (en)

Dried blood spot (DBS) technology is a simple and convenient method for collecting, transporting, and storing blood samples on filter paper, and has numerous applications in the clinical, research, and public health settings. This technique is gaining popularity in the field of forensic science because it facilitates the rapid analysis of prohibited drugs in blood samples and offers significant advantages in toxicology scenarios such as drinking-driving screening, drug abuse detection, and doping detection. However, the lack of a standardized system and the fact that its stability and reliability have not been thoroughly researched and demonstrated limit its application in judicial practice in China. DBS samples can be prepared, stored, and analyzed in various ways, all of which may significantly affect the results. In this study, we developed a method based on ultra-high performance liquid chromatography-tandem mass spectrometry (UHPLC-MS/MS) that focuses on the preparation, pretreatment, analysis, and storage of DBS samples. A thorough investigation was conducted to examine the optimal preparation conditions, including the blood spot matrix, drying technique, and preprocessing parameters, such as the solvent and extraction method. Moreover, the analytical conditions, such as the mobile phase system and elution gradient, were established to facilitate the quantitative detection of methamphetamine, lidocaine, ketamine, fentanyl, and diazepam in both DBS and whole-blood samples. The impact of storage conditions, such as the temperature, humidity, and sealing, on the analytical results of the DBS and whole-blood samples was also examined. The results showed a strong linear relationship for lidocaine and fentanyl within the range of 0.5-100 ng/mL. Similarly, methamphetamine, ketamine, and diazepam exhibited good linearity within the range of 2-100 ng/mL. The coefficients of determination (*r*^2^) ranged from 0.9983 to 0.9997, and the limits of detection ranged from 0.2 to 0.5 ng/mL, indicating a high degree of correlation and sensitivity. Stability tests demonstrated that the five target substances remained stable in the DBS for 60 days, with the measured contents deviating from the nominal values by 15%. Moreover, the measurement results of the DBS samples were highly similar to those of the whole-blood samples, with mean percentage differences of 4.44%, 3.50%, 7.66%, 5.10%, and 5.25% for fentanyl, diazepam, ketamine, lidocaine, and methamphetamine, respectively. Throughout the 60-day storage period, the maintenance of temperatures of -20 and 4 ℃, as well as sealing and dry storage, was not necessary. Room temperature was the most practical storage environment for the DBS samples. The results for each target showed very small concentration differences between the whole-blood and DBS samples, indicating that the DBS samples were suitable for drug and poison analysis in blood. Furthermore, the DBSs exhibited high quantitative consistency with the whole-blood samples, rendering them suitable matrices for preserving blood samples. Because DBS samples are easy to handle and store, they can realize the lightweight preservation of blood samples and provide a novel solution for the analysis and preservation of blood samples in public security practice. We recommend conducting comprehensive validations before utilizing DBS for analysis, particularly in terms of quantification, to ensure the judicial reliability of the results.

根据不同案件性质和追诉期要求,法庭科学领域中最常见的血液检材通常需要在-20 ℃的冷冻室或低温冰柜中长期储存,这使得相关机构承担了较高的维护运营成本^[1]^。此外,检测过程中血液样品的多次冻融很可能会影响物质本身的稳定性^[2]^。干血斑(dried blood spots, DBS)采样是一种使用专用采集卡收集少量血液的采样技术,最初被用于新生儿苯丙酮尿症的筛查^[3]^。随着高灵敏度分析技术的发展,DBS技术现也被广泛应用于临床药物研发、治疗药物监测以及法庭科学等多个领域^[4⇓-6]^。与传统血液检材相比,理论上血液中多数目标物在干血斑中更加稳定^[7,8]^,使用干血斑检材还可显著降低运输、储存过程的设备要求^[9]^。干血斑技术在酒后驾驶检查、滥用药物检测、兴奋剂检测、重金属环境暴露评估等实战场景中具有显著优势^[10⇓⇓⇓-14]^。

但在干血斑样品的制作、储存和分析过程中,存在许多可能对分析结果产生较大影响的因素^[15]^。DBS的面积会随着血液红细胞比容(hematocrit, Hct)的增加逐渐下降^[16]^。不同Hct的血液样品具有不同的黏度,能够对DBS分析的准确度以及分析物的分布状态产生直接影响^[17]^。同时由于分析物结构的差异,分析物在血液扩散时会富集在血斑边缘或者集中在血斑中心。Ren等^[15]^使用放射自显像技术发现DBS样品体积越大,分析物分布不均匀的现象越明显。一些研究人员创新性地使用新型基底尝试减弱血斑基底对目标物回收率、分布均匀性及稳定性的影响^[18]^。Zhang等^[19]^使用二氧化硅涂布纸结合纸喷雾电离技术可以使血斑中的利多卡因(lidocaine)等药物的定量限达到0.1 ng/mL。Damon等^[20]^使用疏水纸基底使血液形成3D干血球体,有效减弱了样品和纸基底之间的相互作用,使地西泮(diazepam)等不稳定有机化合物的稳定性得到提升。然而目前在法庭科学毒物分析领域中,血液和干血斑之间的定量一致性研究较少且目标物在干血斑中的长期储存稳定性没有得到确证,仍缺少规范化的干血斑制作、储存和检测规范。

本研究参考Fan等^[21]^的报道,使用整个DBS进行分析而不是取出部分血斑冲孔,以避免Hct对血斑样品血容量的影响;以分别属于苯丙胺类、可卡因类、苯环利啶衍生物、阿片类和苯二氮卓类药(毒)物的甲基苯丙胺(methamphetamine, MA)、利多卡因、氯胺酮(ketamine)、芬太尼(fentanyl)和地西泮作为目标物,这5种药(毒)物自身稳定性较高,可以有效防止目标物自身不稳定引起降解问题对最终稳定性评估带来误导;结合超高效液相色谱-串联质谱(UHPLC-MS/MS),建立了适用于毒物分析领域的DBS样品的制作方法和前处理方法,考察了温度、湿度和储存密封性对干血斑作为血液样品保存基质的影响,并通过干血斑和全血样本检测结果的对比,验证了干血斑技术的可靠性和准确性。

## 1 实验部分

### 1.1 仪器、试剂与材料

LC-40D型超高效液相色谱仪(日本Shimadzu公司); QTRAP 6500+三重四极杆质谱仪(美国AB Sciex公司),配有电喷雾离子源;Fresco21 高速冷冻离心机(美国Thermo Fisher Scientific公司); VE400电子温控烘箱(德国Memmert公司); KQ-800 KDE型高功率数控超声清洗器(昆山市超声仪器有限公司);PL203电子天平(梅特勒-托利多仪器有限公司)。实验用水由Milli-Q Advantage A10超纯水系统(美国Millipore公司)制备。

甲醇(MeOH)、乙腈(ACN)(均为色谱级,美国Thermo Fisher Scientific公司);甲酸、甲酸铵(均为色谱级,上海麦克林生化科技有限公司); Whatman 903蛋白保存卡(英国Whatman公司); FTA DMPK-A、FTA DMPK-B、FTA DMPK-C采集卡(德国QIAGEN公司);血样采集卡(加强型)、血样采集卡(经典型)、DNA样本采集卡(长春市博坤生物科技有限公司)。

甲基苯丙胺、利多卡因、氯胺酮、芬太尼和地西泮标准溶液(天津阿尔塔科技有限公司)的质量浓度均为1000 μg/mL。氘代甲基苯丙胺( MA-d11)、氘代利多卡因(lidocaine-d10)、氘代氯胺酮(ketamine-d4)、氘代芬太尼(fentanyl-d5)、氘代地西泮(diazepam-d5)标准溶液(天津阿尔塔科技有限公司)的质量浓度均为100 μg/mL。除利多卡因溶于乙腈外,其余物质均溶于甲醇。各标准品的CAS号见附表S1(www.chrom-China.com)。

空白血液委托首都医科大学附属复兴医院对志愿者进行采集。所有志愿者均签署知情同意书且无相关药(毒)物使用史。本项研究已获得公安部鉴定中心科研伦理委员会的批准(2023-13)。

### 1.2 标准溶液配制

标准物质储备液:分别精密吸取甲基苯丙胺、利多卡因、氯胺酮、芬太尼、地西泮标准溶液1 mL,加入9 mL甲醇,配制为100 μg/mL标准物质储备液,于-20 ℃保存;内标物质储备液:分别精密吸取氘代甲基苯丙胺、氘代利多卡因、氘代氯胺酮、氘代芬太尼、氘代地西泮标准溶液1 mL,加入9 mL甲醇,配制为10 μg/mL内标物质储备液,于-20 ℃保存。

5种药(毒)物混合标准储备溶液:精密吸取适量标准物质储备液,以甲醇稀释至质量浓度为10 μg/mL的混合标准储备溶液,于-20 ℃保存;5种药(毒)物内标混合储备溶液:精密吸取适量内标物质储备液,以甲醇稀释至质量浓度为1 μg/mL,于-20 ℃保存。

5种药(毒)物混合标准工作溶液:取适量混合标准储备溶液,以甲醇稀释至质量浓度为1 μg/mL,于-20 ℃保存;5种药(毒)物氘代内标混合工作溶液:取适量内标混合储备溶液,以甲醇稀释至质量浓度为100 ng/mL,于-20 ℃保存。

### 1.3 样品制备

全血样品:取空白全血,加入适量混合标准工作溶液;干血斑样品:准确吸取50 μL全血样品滴在Whatman 903蛋白保存卡上,于60 ℃环境放置20 min。全血和干血斑的质控样品分为低质控(low quality control, LQC)样品、中质控(medium quality control, MQC)样品和高质控(high quality control, HQC)样品3个水平(表1)。

**表 1:** 5种目标物及其内标物的保留时间、质谱参数和质控样品浓度

Compound	Retention time/min	Precursor ion (m/z)	Product ion (m/z)	Declustering potential/V	Collision energy/eV	LQC/ (ng/mL)	MQC/ (ng/mL)	HQC/ (ng/mL)
MA	2.61	150.1	91.0^*^	40	27	5	50	80
			119.0		14			
MA-d11	2.61	161.1	97.0^*^	20	26	-	-	-
			127.1		17			
Lidocaine	2.72	235.1	58.1	40	52	2	50	80
			86.0^*^		23			
Lidocaine-d10	2.72	245.1	64.0	55	55	-	-	-
			96.2^*^		27			
Ketamine	2.75	238.1	125.0^*^	40	39	5	50	80
			207.1		20			
Ketamine-d4	2.75	242.0	129.1*	40	38	-	-	-
			211.0		21			
Diazepam	3.20	285.1	154.0	150	37	2	50	80
			193.0^*^		43			
Diazepam-d5	3.20	290.0	154.1	110	39	-	-	-
			198.2^*^		47			
Fentanyl	3.90	337.2	105.1	80	43	5	50	80
			188.2^*^		30			
Fentanyl-d5	3.90	342.5	105.1	80	40	-	-	-
			188.1^*^		28			

^*^ Quantitative ion. LQC: low quality control; MQC: medium quality control; HQC: high quality control. MA: methamphetamine.

### 1.4 样品前处理

干血斑中目标物的提取:使用打孔器(直径14 mm)取下整个血斑,对半剪开后置于2 mL离心管中,加入500 μL含1 ng/mL氘代内标的甲醇,涡旋振荡10 min(3000 r/min)后取清液经0.22 μm有机膜过滤后待测。

全血中目标物的提取:吸取50 μL全血样品于2 mL离心管中,加入450 μL含1 ng/mL氘代内标的甲醇-乙腈(1:1, v/v),涡旋振荡10 min(3000 r/min),在高速冷冻离心机内离心10 min(12000 r/min)后取上清液经0.22 μm有机膜过滤后待测。

### 1.5 分析条件

色谱条件:Biphenyl 100Å色谱柱(100 mm×3.0 mm, 2.6 μm);流动相:A为5 mmol/L甲酸铵溶液(含0.1%甲酸), B为乙腈;流速:0.4 mL/min;柱温:40 ℃;进样量:5 μL;梯度洗脱程序:0~0.5 min, 10%B; 0.5~3.2 min, 10%B~90%B; 3.2~4.0 min, 90%B; 4.0~4.1 min, 90%B~10%B; 4.1~5.0 min, 10%B。

质谱条件:电喷雾离子源;扫描方式:正离子模式;检测方式:多反应监测(MRM);离子源温度:550 ℃;电喷雾电压:5500 V。5种目标物的质谱参数见表1。

## 2 结果与讨论

### 2.1 血斑制备方法

#### 2.1.1 血斑基底

考察DMPK-A、DMPK-B、DMPK-C采集卡、Whatman 903蛋白保存卡(生产批号分别为7252422W211、7177720W191、7105118W162)、DNA样本采集卡、血样采集卡(加强型)、血样采集卡(经典型)共7种血斑基底对5种目标物的影响,每种血斑基底制备6个平行样品。

安全、经济、简单易行的样品制备方法是干血斑技术发展的必备条件。本研究同时对比了Whatman 903等4种昂贵的进口蛋白保存卡以及3种更经济、更易获得的国产采集卡。以Whatman 903蛋白保存卡中目标物的色谱峰面积为基准,7种基底中各目标物的相对色谱峰面积如图1a所示。DMPK-A和DMPK-B在制作时经过特殊物质处理,具有裂解细胞并使蛋白质变性的能力^[22]^,可能是受其表面化学涂层的影响,甲基苯丙胺、利多卡因、氯胺酮在DMPK-A未检出,芬太尼、利多卡因和地西泮在DMPK-B中的相对峰面积较低(51.69%~60.59%)。Whatman 903、DMPK-C、DNA样本采集卡、经典型和加强型采集卡表面均没有被化学物质浸渍,5种目标物在这几种采集卡上的峰面积均无明显下降。

**图 1:**
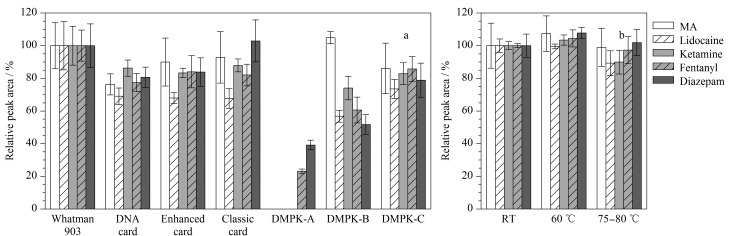
采用不同制作方式时干血斑中目标物的相对峰面积(*n*=6)

以Whatman 903作为血斑基底参考标准,利多卡因受血斑基底类型的影响较大,其在DNA样本采集卡、经典型和加强型采集卡的最大相对峰面积仅为69.03%(DNA样本采集卡),其余目标物均能达到同等的检测效果。甲基苯丙胺、氯胺酮和地西泮在经典型采集卡出现最大相对峰面积,分别为92.84%、87.91%和102.93%。芬太尼在加强型卡出现最大相对峰面积(84.02%)。Whatman 903是经过美国食品药品监督管理局注册认证的采血设备^[23]^,且5种目标物均展现出良好的检测效果,因此Whatman 903被用作后续的方法开发及验证。

由于目标物理化性质的差异,不同类型的纸张可能会对检测产生较大影响,通常不施加任何化学涂层的纯纤维素纸张(Whatman 903、DMPK-C)是制作干血斑的优选材料。然而由于其较昂贵的成本和较差的供货稳定性,DNA样本采集卡、经典型和加强型等国产采集卡可以作为公安基层替代血斑基底,但是在使用时应对基底的影响进行充分考察。

#### 2.1.2 干血斑干燥方式

使用MQC浓度水平的加标血液样品制备干血斑样品,放置于室温、60 ℃和75~80 ℃以考察干燥条件对血斑中目标物的影响,每种温度条件制备6个平行样品。室温条件的血斑样品于通风橱放置4 h并且避免阳光直射;60 ℃条件的血斑样品于烘箱内放置20 min; 75~80 ℃条件的血斑样品于1500 W热风机的气流中放置5 min。

3种干燥条件下各目标物的相对色谱峰面积如图1b所示。以常温干燥的样品为基准,其余两种干燥条件下目标物的相对峰面积为89.28%~107.80%。在75~80 ℃热风下,利多卡因和氯胺酮的相对峰面积略有下降,但仍保持在89.28%、89.92%。对于甲基苯丙胺等具有较强热稳定性的目标物,为发挥干血斑的高通量分析优势,在后续方法开发及验证过程中我们将干血斑于60 ℃环境中放置20 min进行干燥。与Basavaraju等^[24]^的建议相比,本研究大大缩短了样品制备时间。然而需要注意的是,对于稳定性较差的目标物,较高的干燥温度可能会促使其降解^[25]^。因此在应用较高温度加速血斑干燥之前,应该系统地评估目标物的热稳定性。

### 2.2 样品前处理及分析方法优化

#### 2.2.1 提取剂的优化

考察了甲醇、乙腈、甲醇-乙腈(1:1, v/v)、甲醇-水(1:1, v/v)、甲醇(含0.5%甲酸)、乙腈-水(1:9, v/v)6种提取剂对干血斑和全血中目标物的提取效果。当采用乙腈作为提取剂时,甲基苯丙胺未被检出,可能是由于乙腈的低提取效率导致提取液中甲基苯丙胺浓度低于检出限。其余目标物的色谱峰出现明显的峰前沿现象,可能是由于乙腈的洗脱能力强于初始比例流动相,导致出现较强溶剂效应^[26]^。当提取剂为甲醇(含0.5%甲酸)时,由于在酸性环境下血红素与珠蛋白结合较为疏松,血红蛋白会出现明显的血红素脱落现象^[27]^,导致样品提取液呈淡红色;当使用甲醇-水(1:1, v/v)或乙腈-水(1:9, v/v)提取时,由于提取剂中水分比例较高,全血和血斑中部分血液溶解,样品提取液呈深红色浑浊状。使用甲醇-乙腈(1:1, v/v)或甲醇作为提取剂时,样品提取液呈现澄清透明状,各目标物的相对峰面积如图2所示。以采用甲醇作为提取剂时各目标物的色谱峰面积为基准,当采用甲醇-乙腈(1:1, v/v)作为提取剂时,血斑中各目标物的相对峰面积为94.76%~105.84%,全血中各目标物的相对峰面积为94.31%~106.11%。为减少仪器损耗、增强目标物响应,使用甲醇作为血斑样品的提取剂,使用甲醇-乙腈(1:1, v/v)作为全血样品的提取剂进行后续的方法开发及验证。

**图 2:**
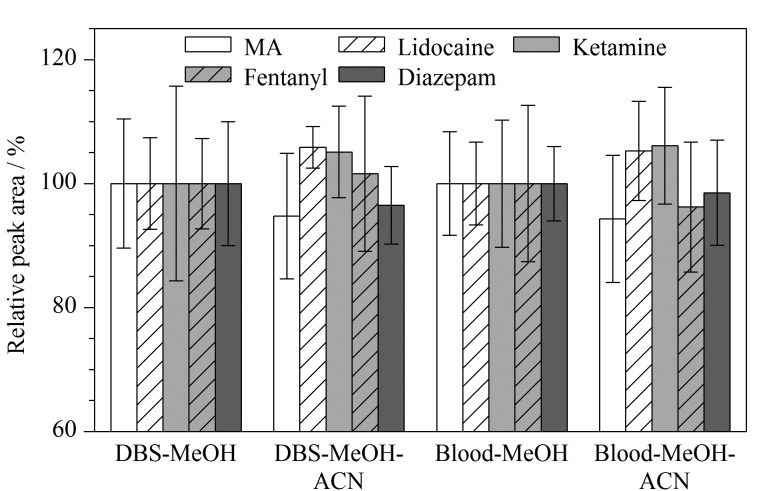
采用不同提取剂时各目标物的相对峰面积(*n*=6)

#### 2.2.2 血斑和全血提取方法优化

考察涡旋振荡(3000 r/min)10 min、超声波(50 ℃恒温水浴)10 min、涡旋振荡(3000 r/min)5 min+超声波(50 ℃恒温水浴)5 min等3种提取方式对干血斑和全血中目标物的提取效果。为适应公安基层现场缺少前处理仪器的现状,还考察了仅将干血斑样品常温下静置10 min的提取效果。

以涡旋振荡10 min时各目标物的色谱峰面积为基准,不同提取方式对血斑和全血中目标物的提取效果如图3所示。在提取方法中引入超声波(50 ℃恒温水浴)对干血斑中芬太尼、地西泮、利多卡因和甲基苯丙胺的提取具有一定的积极作用,但对全血样品中地西泮和甲基苯丙胺具有一定的消极影响。同时经超声波(50 ℃恒温水浴)处理会使采集卡上部分血斑溶解导致提取液呈现淡红色。

**图 3:**
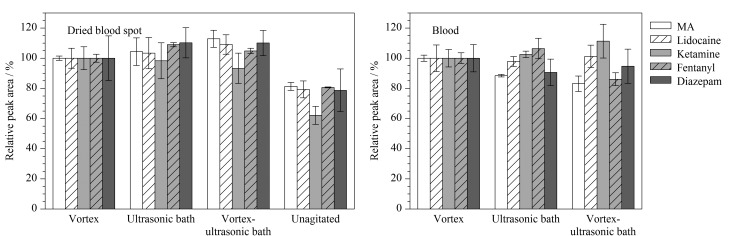
不同提取方法下各目标物的相对峰面积(*n*=6)

相比于全血样品,干血斑样品完全不需要离心。可以发现即使不采取任何提取方法,仅将干血斑样品常温静置时仍能达到涡旋振荡62%~81%的提取效果。干血斑样品对前处理仪器的要求更低,更加适合警务现场处理。为降低前处理仪器的要求,简化样品前处理流程,在后续方法开发及验证过程中使用涡旋振荡10 min作为血斑样品的提取条件,使用涡旋振荡10 min后离心10 min作为全血样品的提取条件。

#### 2.2.3 色谱条件优化

以水(含0.1%甲酸)-乙腈作为流动相,考察CORTECS UPLC T_3_(100 mm×2.1 mm, 1.6 μm)、ACQUITY UPLC BEH C_18_(100 mm×2.1 mm, 1.7 μm)、Biphenyl 100Å色谱柱对各目标物的分离效果;使用Biphenyl 100Å色谱柱,考察水-甲醇、水-乙腈、水(含0.1%甲酸)-甲醇、水(含0.1%甲酸)-乙腈和5 mmol/L甲酸铵水溶液(含0.1%甲酸)-乙腈5种流动相体系对目标物的分离效果。

当使用C_18_和T_3_色谱柱时,甲基苯丙胺出现明显峰前沿现象,利多卡因和氯胺酮具有相同的保留时间,因此选用Biphenyl 100Å色谱柱。对于等浓度的目标物,使用乙腈-水作为流动相时色谱分离效果较好,目标物的信号响应值和稳定性较高;在流动相中加入5 mmol/L甲酸铵和0.1%(v/v)甲酸能够改善目标物峰形并提高目标物的质谱响应;在此条件下5种目标物能够在5 min内有效分离。最终确定5 mmol/L甲酸铵水溶液(含0.1%甲酸)-乙腈作为流动相。在1.5节条件下,各目标物及其氘代内标物的保留时间如表1所示。

#### 2.2.4 质谱条件优化

在多反应监测模式下,依次对去簇电压、碰撞能量、分析物及其内标的前体离子和产物离子等条件进行优化,最终确定的质谱分析条件见表1。

### 2.3 提取回收率和基质效应

以LQC、MQC、HQC共3个浓度水平的质控样品考察提取回收率和基质效应,每个水平制备3个全血或干血斑质控样品。制备LQC、MQC、HQC共3个浓度水平的纯溶剂样品(溶剂为甲醇),每个浓度制备3个样品。将50 μL空白全血或空白干血斑样品按1.4节所述提取,在提取液中添加适量标准品工作液,使最终目标物浓度为LQC、MQC、HQC浓度水平,制备成提取后添加样品,每个浓度水平制备3个样品。

根据质控样品中目标物峰面积与提取后添加样品中目标物峰面积之比评价提取回收率:提取回收率=*B/A*×100%, *A*为提取后添加样品中目标物峰面积,*B*为同浓度水平下质控样品中目标物峰面积。

基质因子(matrix factor, MF)为提取后添加样品中目标物的峰面积与纯溶剂(甲醇)中目标物的峰面积之比。内标归一化基质因子(IS normalized matrix factor, IS-MF)为目标物MF与内标物MF之比^[28,29]^。根据IS-MF和RSD评价基质效应:IS-MF=MF_analyte_/MF_IS_×100%, RSD应小于15%。

血斑样品与全血样品的提取回收率和基质效应结果见表2。干血斑中5种目标物的提取回收率为89.76%~113.55%,基质效应为76.95%~104.81%,RSD为0.47%~5.44%(*n*=3)。全血中5种目标物的提取回收率为86.37%~108.73%,基质效应为83.21%~109.33%,RSD为0.15%~10.75%(*n*=3),均满足方法学要求。

**表 2:** 干血斑和全血样品中5种目标物的提取回收率和基质效应(*n*=3)

Compound	Spiked level	Dried blood spots				Whole-blood				
		Recovery/%	Matrix effect/%	RSD/%		Recovery/%		Matrix effect/%		RSD/%
MA	LQC	96.19	91.01	4.29		107.36	83.21		1.24	
	MQC	113.55	79.06	3.03		89.95	84.63		2.50	
	HQC	103.08	87.63	1.63		97.31	90.87		2.00	
Lidocaine	LQC	102.12	104.09	2.74		99.52	93.46		1.70	
	MQC	102.53	77.54	4.65		86.79	87.15		5.85	
	HQC	91.43	87.14	4.21		91.78	85.61		4.97	
Ketamine	LQC	89.76	88.29	0.47		87.67	101.25		0.56	
	MQC	97.34	81.07	1.60		89.27	109.33		2.67	
	HQC	93.90	91.13	4.13		86.37	100.33		1.56	
Fentanyl	LQC	104.88	104.81	2.94		108.73	108.66		0.65	
	MQC	111.22	87.22	5.44		92.65	86.00		0.81	
	HQC	97.32	94.20	1.88		96.31	97.96		0.15	
Diazepam	LQC	94.31	87.61	4.94		106.18	86.33		0.24	
	MQC	104.72	76.95	3.62		104.24	88.71		2.42	
	HQC	106.80	85.17	5.01		107.40	94.52		10.75	

### 2.4 方法学验证

参照国家质量监督检验检疫总局、中国国家标准化管理委员会联合发布的GB/T 27417-2017《合格评定化学分析方法确认和验证指南》^[30]^以及美国食品药品监督管理局发布的“bioanalytical method validation guidance for industry”^[31]^进行方法学实验的设计与验证。干血斑和全血分析的方法验证考虑了以下参数:检出限(limit of detection, LOD)、定量限(limit of quantification, LOQ)、校准曲线、精密度、准确度。前处理方法和实验条件均按1.4节和1.5节所述。

#### 2.4.1 检出限、定量限与校准曲线

按1.3节所述方法制备质量浓度为0.2、0.5、1、2、5、10、20、50和100 ng/mL的全血及干血斑校准样品,并按照1.4节所述方法处理后进行UHPLC-MS/MS分析。每个质量浓度的校准样品制作5份平行样品并在不同批次进行分析。以目标物与对应内标的定量离子对峰面积比值(*y*)为纵坐标,目标物的质量浓度(*x*, ng/mL)为横坐标拟合标准曲线(权重:1/*x*)。检出限的信噪比大于3,定量限的信噪比大于10。

利多卡因和芬太尼的线性范围为0.5~100 ng/mL,甲基苯丙胺、氯胺酮、地西泮的线性范围为2~100 ng/mL,决定系数(*r*^2^)为0.9983~0.9997,满足方法学要求。结果见表3。

**表 3:** 干血斑和全血中5种目标物的线性范围和校准曲线

Compound	LOD/ (ng/mL)	LOQ/ (ng/mL)	Linear range/ (ng/mL)	Calibration curve	Coefficient of determination (r^2^)
MA	0.5	2	2-100	DBS: y=0.28675x+0.06452	0.9994
				blood: y=0.25514x+0.00928	0.9983
Lidocaine	0.2	0.5	0.5-100	DBS: y=0.18878x+0.04239	0.9996
				blood: y=0.17963x+0.01635	0.9994
Ketamine	0.5	2	2-100	DBS: y=0.26189x+0.05804	0.9996
				blood: y=0.28151x+0.01546	0.9993
Fentanyl	0.2	0.5	0.5-100	DBS: y=0.28675x+0.06452	0.9990
				blood: y=0.30969x+0.03796	0.9997
Diazepam	0.5	2	2-100	DBS: y=0.14477x+0.06180	0.9993
				blood: y=0.14490x+0.02882	0.9985

*y*: ratio of peak area between the analyte and its internal standard; *x*: mass concentration, ng/mL; weight: 1/*x*.

#### 2.4.2 精密度和准确度

以LOQ、LQC、MQC、HQC共4个浓度水平的质控样品进行精密度和准确度考察。每个水平独立制备5份全血样品和5份干血斑样品。每天测定一个批次,连续测定3天。精密度以RSD表示,准确度以偏倚(bias,即测定平均值和参考值的差值与参考值的百分比)表示。

干血斑样品与全血样品的精密度和准确度结果见表4。在干血斑样品中各目标物的日内精密度范围为1.24%~5.96%,日间精密度范围为2.14%~9.86%,准确度范围为-7.75%~10.10%。在全血样品中各目标物的日内精密度范围为1.47%~5.32%,日间精密度范围为1.57%~5.93%,准确度范围为-6.74%~10.07%,均满足方法学要求。

**表 4:** 干血斑和全血样品中5种目标物的精密度和准确度偏差(*n*=5)

Compound	Spiked level	DBS				Whole-blood			
		RSDs/%		Bias/%		RSDs/%			Bias/%
		Intra-day	Inter-day		Intra-day		Inter-day		
MA	LOQ	3.62	8.95	0.26		5.03	5.93	6.27	
	LQC	4.53	5.15	-1.75		5.32	4.53	-3.81	
	MQC	2.80	5.48	1.30		3.39	3.27	-2.83	
	HQC	5.51	5.64	-7.39		3.05	3.06	0.67	
Lidocaine	LOQ	5.96	5.46	10.10		4.38	4.12	5.19	
	LQC	1.79	5.04	2.66		1.47	1.57	4.68	
	MQC	1.45	2.58	0.09		1.85	2.30	-2.50	
	HQC	3.86	4.48	-5.00		1.90	2.06	-5.95	
Ketamine	LOQ	1.89	8.65	4.30		4.11	4.54	10.07	
	LQC	3.39	4.59	-1.06		3.53	4.09	-2.74	
	MQC	1.45	3.82	-0.30		4.05	3.75	2.89	
	HQC	1.73	5.33	-7.75		2.00	2.06	1.62	
Fentanyl	LOQ	1.24	2.14	-6.36		4.86	4.23	1.78	
	LQC	2.39	6.97	5.08		4.24	4.58	4.77	
	MQC	1.67	3.21	3.31		2.15	2.04	0.04	
	HQC	4.05	4.17	-3.41		3.25	3.39	-4.00	
Diazepam	LOQ	3.66	9.86	5.27		3.69	4.46	7.04	
	LQC	3.11	4.95	0.63		1.58	1.63	-6.74	
	MQC	4.41	5.64	3.75		3.37	3.04	2.86	
	HQC	3.95	5.88	-1.75		3.95	4.42	1.54	

### 2.5 干血斑样品的储存稳定性

将2个浓度水平(LQC、HQC)的DBS质控样本在不同条件下储存(表5)。在储存第0、1、2、5、7、14、21、31、60天时测定干血斑样品中的信号响应(目标物与对应内标的峰面积比值,每次测定6个储存样本),并与新制备质控样品中目标物的信号响应进行比较。密封条件(sealed)的样品袋内均放置硅胶干燥剂和湿度指示卡以保持低湿度环境。

**表 5:** 干血斑样品储存条件

Sample No.	Temperature/℃	Relative humidity/%	Seal
S1	25-28 (RT)	40	unsealed
S2	25-28 (RT)	10	sealed
S3	4	40	unsealed
S4	4	10	sealed
S5	-20	40	unsealed
S6	-20	10	sealed

RT: room temperature.

在考察的60天内,DBS样品中的5种目标物在6种储存条件下均显示出良好的稳定性(见附表S2~S6)。在低、高两个浓度水平下,各目标物的测定含量与理论值的偏差均在15%以内^[32]^。以干血斑中芬太尼为例,其测定含量变化如图4所示。在60天的监测时间内芬太尼测定含量比值平均为102.34%,最高值为113.06%,最低值为86.53%。即使在室温、40%湿度条件下,干血斑中的芬太尼也能稳定保持60天。DBS不必保存在-20 ℃和4 ℃环境中,室温显然是最方便的存储条件并且不需额外的干燥设施。DBS的易采样性、易储存性和DBS中目标物的高稳定性是DBS技术的关键优势。在保存过程中,干血斑中的酶和蛋白质处于失水状态而失活,化合物的转化、降解等化学反应受到抑制,使得化合物更加稳定。DBS能够在常温下稳定储存的优势特别适用于检材的异地运输和检测。当采样地点远离实验室或取样后需要几天时间才能进行分析时,DBS不需要大容量的冷冻室,维护成本显著降低,是一种可靠的检材储存形式。

**图 4:**
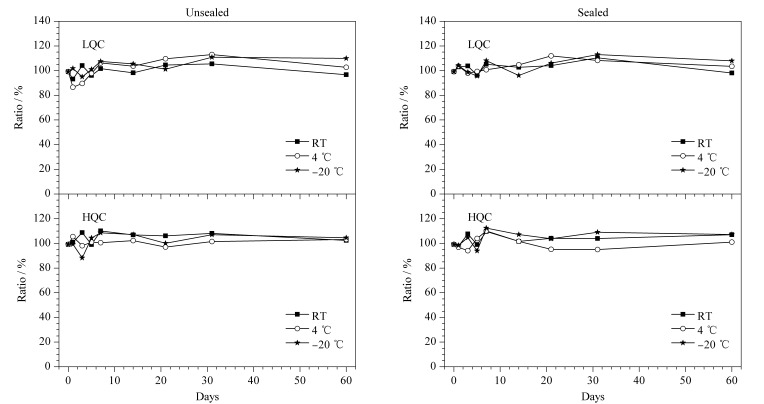
60天内不同储存条件下储存样品与质控样品中芬太尼的含量比

### 2.6 干血斑和全血样品的定量一致性

使用已建立的干血斑和全血分析方法,分别在2、5、50、80 ng/mL 4个浓度水平下(每个水平制备12个平行样本)进行干血斑和全血中目标物的定量一致性考察。使用Spearman秩相关系数*ρ*和Passing-Bablok回归分析法评估检测方法的相关性,使用Bland-Altman散点图评估检测方法的一致性^[32]^。

两种形态血液样品的定量准确度箱型图见图5。DBS中5种目标物的测量结果与全血相近。仅在一例血液样品中利多卡因准确度偏差高于15%,其余干血斑样品和全血样品的检测准确度在85.46%(地西泮)和114.95%(芬太尼)之间,RSD均<15%。Spearman秩相关系数是适用于非正态分布数据的检测方法。DBS和全血样本间5种分析物的Spearman秩相关系数*ρ*=0.921~0.955,说明DBS和全血样本间存在很强的相关关系。不过检测结果的强相关性并不一定意味着两种检测方法有良好的定量一致性,这种方法无法反映出系统误差并且易受异常值的影响。因此有必要使用Passing-Bablok回归分析和Bland-Altman图确定检测异常值和系统误差^[33]^。

**图 5:**
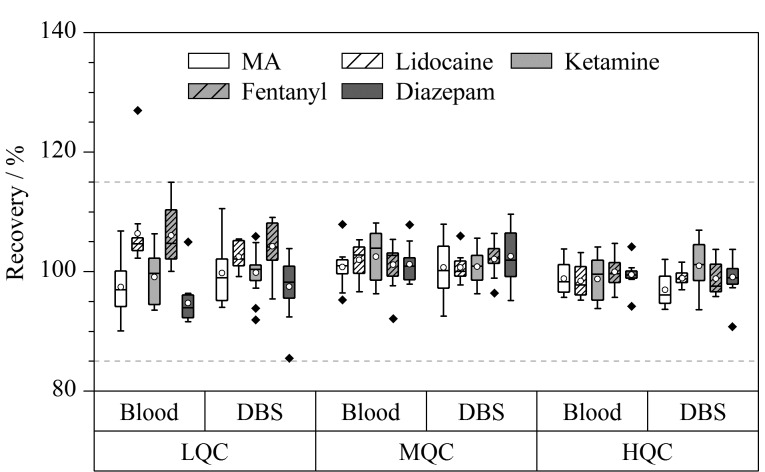
干血斑中5种目标物的定量准确度(*n*=12)

Passing-Bablok回归分析和Bland-Altman图是定量分析和定性分析的结合,能够同时控制系统误差和随机误差,常用于判断两种方法测定结果的等效性^[32]^。以芬太尼为例,干血斑和全血样品的检测结果展示在图6。Passing-Bablok回归结果显示,DBS和全血中各目标物的结果数据没有显著的系统误差(截距=-0.06~0.02 ng/mL)和比例误差(斜率=0.99~1.02)。以干血斑和全血样本测定结果的差值为*Y*变量,以两者测定结果的均值为*X*变量,绘制Bland-Altman散点图,并将*Y*均值±1.96×SD的两条直线作为参考线。如果散点在*Y*=0水平线的两侧均匀分布且大部分散点分布在一致性限度(limits of agreement, LOA)范围内,说明两种方法具有较高的一致性。

**图 6:**
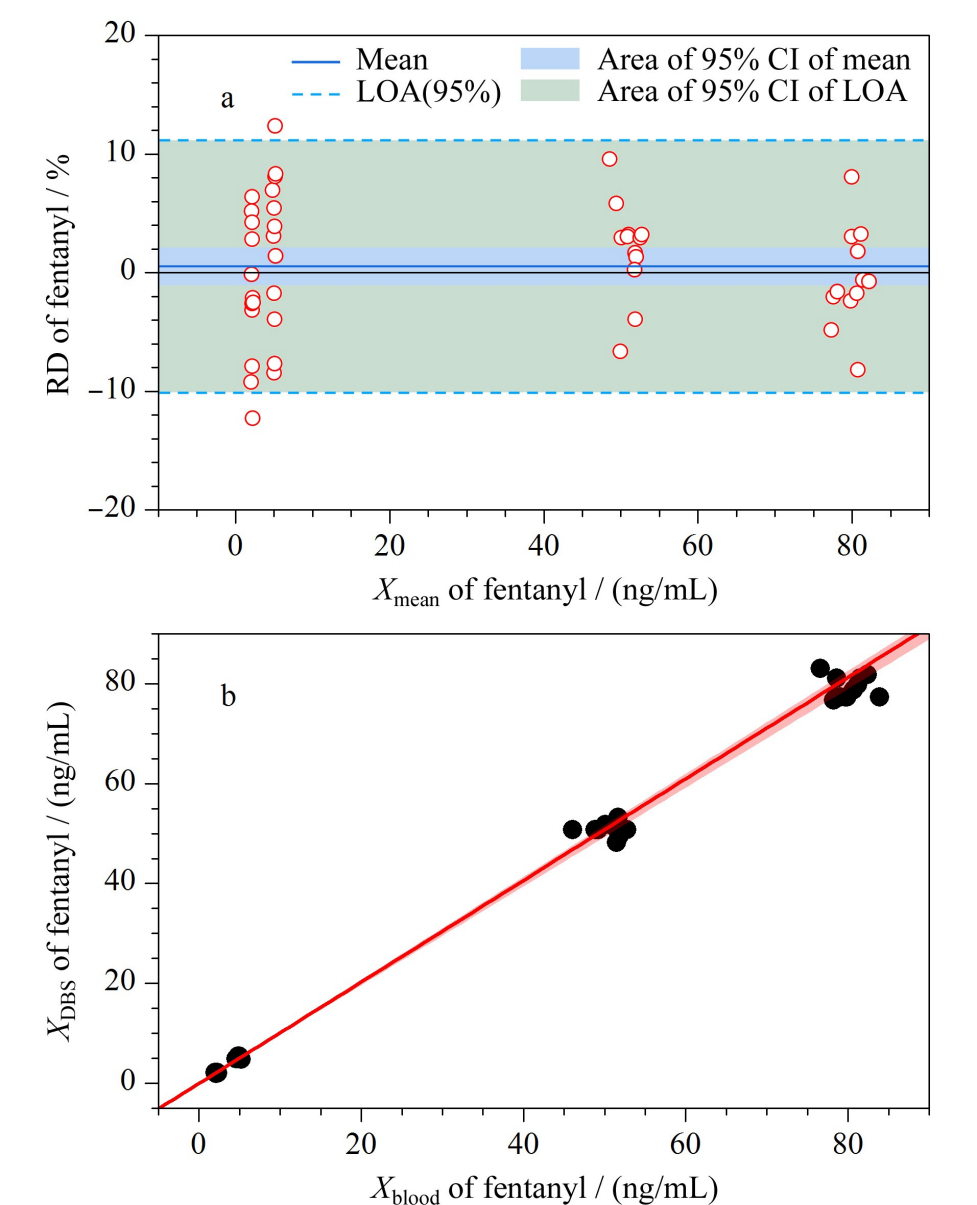
(a)全血和DBS之间芬太尼的浓度差异与(b) DBS中芬太尼浓度与全血浓度的Passing-Bablok分析

以相对偏差(relative difference, RD)反映DBS样本和对应的全血样本的差异:RD=|*X*_DBS_ -*X*_blood_*|/X*_mean_×100%,其中*X*_mean_等于(*X*_blood_+*X*_DBS_)/2, *X*_blood_为全血样本中目标物的质量浓度,*X*_DBS_为DBS样本中目标物的质量浓度。Bland-Altman图显示,DBS样本和对应的全血样本间芬太尼、地西泮、氯胺酮、利多卡因和甲基苯丙胺测量浓度的相对偏差分别为4.44%、3.50%、7.66%、5.10%和5.25%。全血和DBS之间的目标物浓度均在20%的误差接受标准内^[32]^。因此,DBS适合血液中这5种毒物的定量分析,与全血样品具有较高的检测一致性。

## 3 结论

本研究通过综合因素的系统性考察,建立了一种简便快捷的干血斑制作和储存方法,形成了一个标准化的干血斑制作、分析和储存指南,方法简便,易于操作。以甲基苯丙胺、利多卡因、氯胺酮、芬太尼和地西泮作为目标物,通过前处理方法优化和考察,确定60 ℃作为干燥温度,甲醇作为提取剂,涡旋振荡作为提取方式,使整个血斑制作和预处理程序都能够在20 min内完成。5种药(毒)物在干血斑中的储存稳定性好,定量结果与全血样品中各目标物的结果相比没有显著的系统误差和比例误差,具有较高的一致性。在60天的保存期内,室温显然是最方便的DBS存储环境,-20 ℃和4 ℃的温度以及密封干燥并不是必备储存条件。

干血斑样品检测便捷且易于保存,能够实现血液样品保存的轻量化和规范化,为公安实践工作中分析、保存血液检材提供了新方案。然而由于目标物性质的差异,血斑基底、干燥方式、提取剂、提取方式、仪器分析条件、储存条件等会对分析结果产生较大影响。作为一种新型的毒物分析样品形态,在使用干血斑进行分析前特别是涉及定量问题时,应进行完整的验证程序,包括准确度、精密度、检出限、定量限、回收率、基质效应以及稳定性等一般生物分析方法所要求的内容。此外还应评估目标物和DBS在不同条件下的稳定性,特别是在尝试使用较高温度加速DBS干燥时,必须评估目标物的热稳定性。
